# Detection of SXT/R391 integrative conjugative elements carrying tigecycline resistance genes in *Shewanella* spp. isolated from retail seafood

**DOI:** 10.1128/aac.01742-24

**Published:** 2025-06-26

**Authors:** Wenhui Zhang, Kai Peng, Ming Liu, Xuesong Luo, Zhiqiang Wang, Ruichao Li

**Affiliations:** 1 Jiangsu Co-Innovation Center for Prevention and Control of Important Animal Infectious Diseases and Zoonoses, College of Veterinary Medicine, Yangzhou University 614704, Yangzhou, China; 2 Institute of Comparative Medicine, Yangzhou University 614704, Yangzhou, Jiangsu, China; 3 College of Nursing and Health Management & College of Life Science and Chemistry, Wuhan Donghu College 419882, Wuhan, China; 4 National Key Laboratory of Agricultural Microbiology, Huazhong Agricultural University 124443, Wuhan, China; Universita degli Studi di Roma "La Sapienza", Rome, Italy

**Keywords:** tigecycline resistance, *Shewanella*, tet(X4), *tmexCD2-toprJ2*, ICEs

## Abstract

Tigecycline is a last resort antibiotic that is used to treat serious infections; however, some bacteria have developed tigecycline resistance by producing a tigecycline-inactivating enzyme or tigecycline resistance efflux pump, encoded by *tet*(X) and *tmexCD-toprJ* genes, respectively. Tons of seafood are consumed annually in China; however, whether seafood harbors tigecycline-resistant bacteria is not known. In this study, we isolated various tigecycline-resistant bacteria from retail seafood; among these, *Shewanella* was the predominant tigecycline-resistant genus (33/76, 43.4%). Genomic sequencing revealed that two *Shewanella* strains carried the *tet*(X4) gene, while one *Shewanella chilikensis* strain co-harbored *tmexCD2-toprJ2* and *bla*_NDM-1_ genes. The *tet*(X4) and *tmexCD2-toprJ2* were found to be located on novel members of the SXT/R391 family of integrated conjugative elements (ICEs). As per our knowledge, this is the first report on the emergence of SXT/R391 ICEs carrying *tet*(X4) or *tmexCD2-toprJ2* gene in *Shewanella* strains. The SXT/R391 family ICEs could mediate the spread of tigecycline resistance genes among aquatic bacteria, and contact between seafood and consumers may lead to the dissemination of tigecycline-resistant bacteria. Our study revealed that *Shewanella* spp. may act as potential reservoirs of tigecycline resistance genes.

## INTRODUCTION

Tigecycline belongs to the glycylcycline class of broad-spectrum antimicrobials. It is considered a last-resort antibiotic for the treatment of severe infections caused by multidrug-resistant (MDR) pathogens, especially carbapenem-resistant pathogenic bacteria (1). However, tigecycline resistance genes, such as *tet*(X) and *tmexCD-toprJ*, as well as their variants, have emerged and disseminated among Gram-negative bacteria. To understand the mechanism by which tigecycline resistance genes evolved and disseminated in bacteria, previous studies characterized tigecycline-resistant bacteria, isolated from diverse environments, such as soil, freshwater, freshwater fishes, livestock and poultry farms, retail meats, and hospitals and hospital wastewater (2–5).

China produces over 60 million metric tons of seafood and consumes more than 50 million metric tons of seafood annually. Seafood is recognized as a significant reservoir of antibiotic-resistant bacteria. For example, a *Citrobacter meridianamericanus* strain and nine *Escherichia coli* strains, isolated from fishes, clams, and crabs, were found to harbor plasmids containing the *tet*(X4) gene (6). However, it is widely acknowledged that *E. coli* and *C. meridianamericanus* are not indigenous to aquatic environments (7). Therefore, the presence of tigecycline-resistant aquatic bacteria in retail seafood in China remains unknown.

The *Shewanella* genus comprises gram-negative, facultative anaerobic bacteria that primarily inhabit aquatic environments (8–10). In recent years, there has been a significant rise in *Shewanella* infections worldwide. Several *Shewanella* species, such as *Shewanella algae*, *Shewanella putrefaciens*, and *Shewanella xiamenensis*, have been found to be associated with humans (11, 12) and are considered to be opportunistic human pathogens. In addition, *Shewanella* species carry several antibiotic resistance genes, including carbapenem, beta-lactam (13, 14), and the variants of colistin (*mcr*) resistance genes (15). However, to date, only the *S. xiamenensis* strain, isolated from a drainage sample in Vietnam, has been reported to carry the tigecycline resistance gene *tet*(X4) (16).

SXT/R391 integrated conjugative elements (ICEs) shared a nearly identical integrase Int, which was considered to mediate their site-specific integration into the 5′ end of the *prfC* gene in the host chromosome. Previously, studies have revealed that SXT/R391 family ICEs contain 52 nearly conserved core genes. In addition, four variable regions (VRI–IV) and five hotspots (HS1–5) have been reported, which are comprised of variable genes conferring a variety of functions including antibiotic resistance. Several studies have investigated the presence of the SXT/R391 ICE family members in a variety of bacterial genera, including Vibrio (17), *Proteus* (18), *Providencia* (19), *Marinomonas* (20), and *Shewanella* (21). Among the SXT/R391 ICE members, identified in *Proteus* spp., *Pseudomonas aeruginosa*, and *Providencia rettgeri*, only a few were found to carry tigecycline resistance genes, like *tet*(X6) or *tmexCD3-toprJ1b* (22–27). Therefore, further research is required to identify the presence of SXT/R391 ICEs harboring tigecycline resistance genes in other bacterial genera.

To investigate the prevalence of tigecycline-resistant bacteria in seafood, we surveyed raw retail seafood sold in the supermarkets of seven cities, namely Wuhan, Baise, Qingdao, Nanjing, Dandong, Nantong, and Shanghai, in China, from 2021 to 2022. We isolated several tigecycline-resistant bacteria belonging to different genera, including *Shewanella*, *Proteus*, *Morganella*, *Providencia*, *Pseudomonas*, *Pseudoalteromonas*, *Aeromonas*, *Citrobacter*, *Raoultella*, *Kluyvera*, *Enterobacter*, *Myroides*, *Janthinobacterium*, and *Serratia*. Among these, the majority of tigecycline-resistant bacteria belonged to the genus *Shewanella*. We identified two *Shewanella* strains that carried the *tet*(X4) gene and one *Shewanella* strain that co-harbored the *tmexCD2-toprJ2* and *bla*_NDM-1_ genes. Whole genome sequencing (WGS) revealed that the *tet*(X4) and *tmexCD2-toprJ2* genes were located in the SXT/R391 ICE family. The *tmexCD2-toprJ2*-containing *Shewanella* species also possessed a novel *Salmonella* genomic island 1 (SGI1) variant carrying the *bla*_NDM-1_ gene. We also isolated eight *Shewanella* isolates that did not harbor the *tet*(X) or *tmexCD-toprJ* gene but were resistant to tigecycline; however, this phenotype was not transferable. Our results suggest that retail seafood carries diverse tigecycline-resistant bacteria and that *Shewanella* spp. are the predominant tigecycline-resistant bacteria in seafood.

## MATERIALS AND METHODS

### Isolation and identification of tigecycline-resistant bacterial species

For this analysis, we obtained raw seafood from supermarkets in Wuhan, Baise, Qingdao, Nanjing, Dandong, Nantong, and Shanghai, in China, during 2021 and 2022. Each sample was packaged individually in a sterile bag and placed in an ice box until further analysis. For pretreatment, the sample was treated with 9 mL of 2% NaCl-supplemented Luria-Bertani (LB) medium (LBS) containing 4 µg/mL tigecycline and homogenized in a tissue homogenizer for 3 minutes. Thereafter, 1 mL of the supernatant was transferred to a fresh tube along with LBS broth supplemented with 4 µg/mL tigecycline and cultured for 10 hours at 30°C. The culture was streaked on LBS agar plates containing 4 µg/mL tigecycline and incubated for 10–14 hours at 30°C. Different colonies obtained on the LBS agar plates were purified and subsequently screened for the presence of *tet*(X) and *tmexCD-toprJ* genes by polymerase chain reaction (PCR) using specific primers (Table S4). Matrix-assisted laser desorption/ionization-time of flight (Bruker) or 16S rDNA sequencing was used to identify the taxonomy of the bacterial isolates.

### Antimicrobial susceptibility testing

The broth microdilution method was used to determine the susceptibility of *Shewanella* isolates and transconjugants to 16 antimicrobials, namely meropenem, imipenem, tigecycline, colistin, ceftiofur, ciprofloxacin, chloramphenicol, tetracycline, kanamycin, gentamicin, streptomycin, ceftriaxone, aztreonam, amikacin, ampicillin, and amoxicillin-clavulanic acid. The results were interpreted according to the guidelines provided in “non-Enterobacterales” in the Clinical and Laboratory Standards Institute (2020) (28). The breakpoints of tigecycline and colistin were interpreted according to the European Committee on Antimicrobial Susceptibility Testing v12.0 criteria (http://www.eucast.org/clinical_breakpoints/). The *E. coli* ATCC strain 25922 served as the quality control sample.

### Extraction, WGS, and assembly of tigecycline-resistant *Shewanella* isolates

Genomic DNA of tigecycline-resistant *Shewanella* isolates was extracted using the FastPure Bacteria DNA Isolation Mini Kit (Vazyme, China). Briefly, the bacterial sample was centrifuged at 13,500 *× g* for 1 minute, and 2 mL of the bacterial culture was collected and treated with proteinase K and RNase. The genomic DNA was extracted using the column extraction method, and its quality and concentration were evaluated by gel electrophoresis and the Colibri LB spectrophotometer (Titertek-Berthold, Germany), respectively. The whole genomes of *Shewanella* isolates were sequenced by the Illumina Hiseq 2500 platform (Illumina) using two-paired libraries with 150 bp average length and 150 × coverage. The long-read Oxford Nanopore Technologies (ONT) MinION platform was employed to further sequence the genomes of *Shewanella* isolates harboring *tet*(X4) or *tmexCD-toprJ* genes. The Illumina read data were assembled using SPAdes v3.13.1 at default parameters (29). The hybrid *de novo* assembly of ONT reads was generated using Unicycler v0.4.8 (30).

### Phylogenetic analysis of *Shewanella* isolates and SXT/R391 ICEs

The phylogenetic relationships of the isolated *Shewanella* strains were determined using the whole genome sequences of representative *Shewanella* strains available in the NCBI database (https://www.ncbi.nlm.nih.gov/genbank/). Information on these genome sequences is provided in Table S2. The draft genomes were annotated using Prokka v1.12, and the phylogenetic tree was built using Roary and FastTree based on single-nucleotide polymorphisms (SNPs) in the core genomes (31–33). The distances of SNPs in the core genomes were analyzed using snp-dists v0.7.0 (https://github.com/tseemann/snp-dists). Phylogenetic analysis of the isolated SXT/R391 ICEs and representative ICEs from the NCBI database was performed based on the SNPs in the conserved core genes. Relevant information on the representative ICEs is provided in Table S3. Phylogenetic trees were constructed using the MEGA 11.0 software via the maximum-likelihood method with 1000 replications of bootstrap values (34). The information on conserved core genes used in the phylogenetic trees is provided in Table S5.

### Comparative analysis and genetic characterization of the novel SXT/R391 ICEs

The complete genome sequences of *Shewanella* isolates harboring SXT/R391 ICEs were annotated through the Rapid Annotation using the Subsystems Technology annotation server (http://rast.nmpdr.org/) (35). ResFinder v4.1, PlasmidFinder v2.1, and ISfinder v2.1 were used to identify ARGs, plasmid replicons, and insertion sequences, respectively. The novel SXT/R391 ICEs, identified in this study, were compared with the representative ICEs available in the public database (https://www.ncbi.nlm.nih.gov/genbank/). The conserved and variable regions in novel ICEs were compared with those in representative ICEs, and the genetic context of the tigecycline resistance genes was illustrated. Easyfig v2.2.3 was used to visualize the SXT/R391 ICEs (36).

### Conjugation assays

The transferability of tigecycline resistance was determined through mating assays using rifampicin-resistant *E. coli* C600 as the recipient. Overnight cultures of the donor strain and *E. coli* C600 were mixed in a 1:1 ratio and incubated overnight at 37°C. The culture was then serially diluted by 10 folds and plated onto selective LB agar plates containing 300 µg/mL rifampicin and 4 µg/mL tigecycline, to count transconjugants. Simultaneously, 10-fold serially diluted cultures were plated onto LB agar plates containing 300 µg/mL rifampicin to count the recipient cells. The plates were incubated overnight at 37°C, and colony-forming units were recorded for three independent replicates. The transfer frequency of ICEs was determined by calculating the number of transconjugants per recipient cell.

### Pairwise growth competition assay

For this assay, overnight cultures of the NJT6 or NTT9 (SXT/R391 ICE-bearing *E. coli* C600) transconjugants and *E. coli* C600 were diluted to 0.5 McFarland standard and mixed at 1:1 ratio in 5 mL of LB broth. The mixtures were incubated at 37°C for 96 hours with shaking, and 5 µL of culture was reinoculated in 5 mL of fresh LB broth every 24 h. The bacterial cultures were serially diluted by 10-fold and inoculated on LB agar plates in the presence or absence of 4 µg/mL tigecycline. The relative fitness was calculated as follows: w = ln(NRt/NR0)/ln(NSt/NS0), where NR is the number of resistant clones and NS is the number of susceptible clones, with values < 1 indicating the fitness cost.

## RESULTS AND DISCUSSION

### Sample collection and bacterial identification

In this study, we isolated various bacterial species belonging to *Proteus* (*n* = 2), *Morganella* (*n* = 8), *Providencia* (*n* = 3), *Pseudomonas* (*n* = 4), *Pseudoalteromonas* (*n* = 4), *Aeromonas* (*n* = 7), *Citrobacter* (*n* = 6), *Raoultella* (*n* = 2), *Kluyvera* (*n* = 3), *Enterobacter* (*n* = 1), *Myroides* (*n* = 1), *Janthinobacterium* (*n* = 1), *Serratia* (*n* = 1), and *Shewanella* (*n* = 33) from retail seafood samples. All the bacterial isolates were screened to detect the presence of *tet*(X) and *tmexCD-toprJ* genes using PCR amplification. Except for *S. algae* NJT6 and *Shewanella chilikensis* NTT9 carrying *tet*(X) and *S. chilikensis* ST10 encoding *tmexCD-toprJ*, other isolates did not harbor *tet*(X) or *tmexCD-toprJ* gene. The strains NTT9 and NJT6 were isolated from *Ruditapes philippinarum* (manila clams) purchased from Nantong and Nanjing cities, respectively. The strain ST10 was isolated from *Sinonovacula constricta* (Chinese razor clam) purchased from Nanjing city. Notably, no *E. coli* strains were isolated from the seafood samples in our analysis, unlike a previous study (6). It is likely that the addition of NaCl in the LBS medium inhibited the growth of *E. coli* in our analysis.

### Resistance phenotype of tigecycline-resistant *Shewanella* isolates

As *Shewanella* strains were predominant (33/76, 43.4%) among all the seafood bacterial isolates, we examined whether these strains were resistant to tigecycline. For this analysis, we randomly chose 10 *Shewanella* strains, in addition to strains NJT6, NTT9, and ST10. Antimicrobial susceptibility testing showed that all 13 *Shewanella* isolates were MDR strains (Table S1), with similar antibiotic resistance profiles. They exhibited high resistance to tigecycline (*n* = 13/13, 100%), tetracycline (*n* = 13/13, 100%), chloramphenicol (*n* = 13/13, 100%), streptomycin (*n* = 12/13, 92.3%), ampicillin (*n* = 12/13, 92.3%), ciprofloxacin (*n* = 11/13, 84.6%), and aztreonam (*n* = 8/13, 61.5%). Additionally, these isolates exhibited higher MIC values for imipenem compared to meropenem, but all the isolates were susceptible to amikacin and gentamicin.

**Fig 1:**
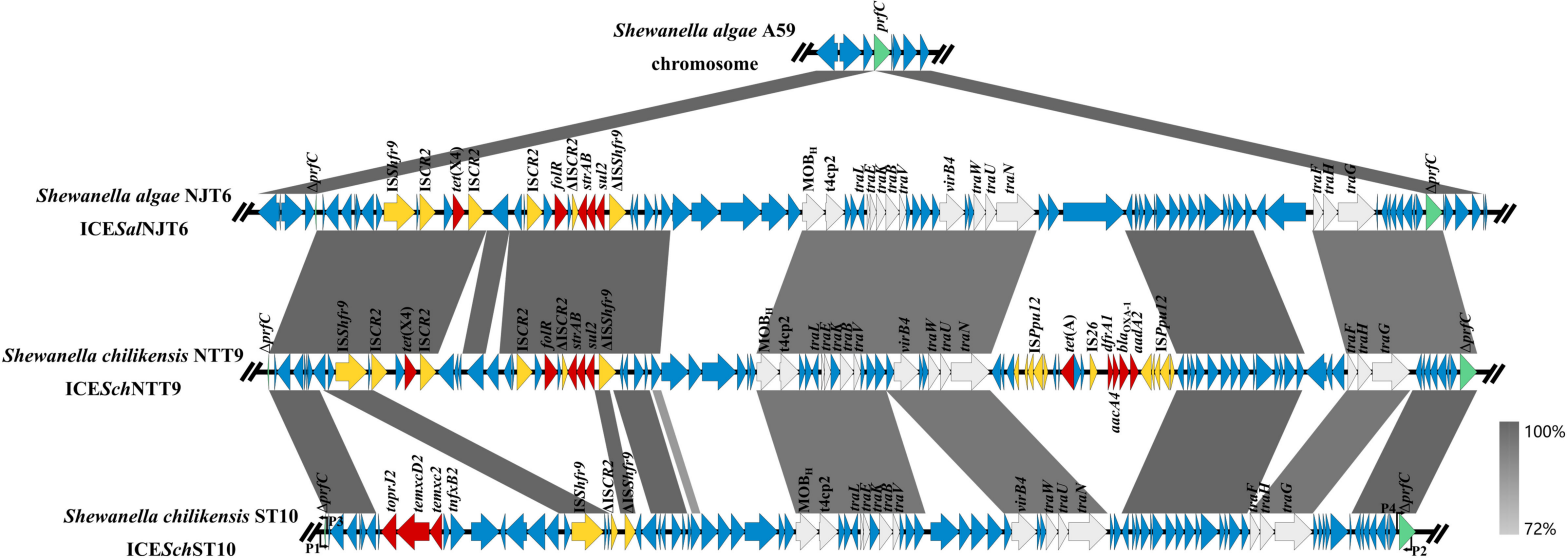
Genomic map of the ICEs identified in this study. The red and yellow arrows represent the drug resistance genes and insertion sequences, respectively. T4SS-encoding genes were marked by white color, and the insertion site-*prfC* genes are highlighted in green. Primers P1, P2, P3, and P4 designed to identify the circular form of ICE*Sch*ST10 were marked as black arrows.

We investigated the genomic structures of *tet*(X) or *tmexCD-toprJ* genes in the strains NJT6, NTT9, and ST10 by WGS. The sequencing results revealed that the tigecycline resistance genes in strains NJT6, NTT9, and ST10 were located in ICEs, designated as ICE*Sal*NJT6, ICE*Sch*NTT9, and ICE*Sch*ST10, respectively (Fig. 1; Table 1). Upon comparison with the genome sequence of *S. algae* A59, we found that ICE*Sal*NJT6, ICE*Sch*NTT9, and ICE*Sch*ST10 were integrated into the 5′ end of the *prfC* gene (which encodes peptide chain release factor three involved in translational regulation) in these *Shewanella* genomes.

**TABLE 1:** Characteristics of the SXT/R391 ICEs and ICE-harboring strains detected in this study

ICE	Strain	Species	Host	Year and location of isolation	Resistance genes in bacterial isolates	Resistance genes in ICE
ICE*Sal*NJT6	NJT6	*Shewanella algae*	*Ruditapes philippinarum*	Nanjing city, Jiangsu Province, China, 2021.07	*qnrA3*, *bla*_OXA-29_, *sul2*, *strA*, *strB*, *floR*, *tet*(A)*, tet*(X4)	*sul2*, *strA*, *strB*, *floR*, *tet*(X4)
ICE*Sch*NTT9	NTT9	*Shewanella chilikensis*	*Ruditapes philippinarum*	Nantong city, Jiangsu Province, China, 2021.07	*qnrA1*, *bla*_OXA-960_, *tet*(X4), *floR*, *strA*, *strB*, *tet*(A), *dfrA1*, *aacA4*, *bla*_OXA-1_, *aadA2*	*tet*(X4), *floR*, *strA*, *strB*, *tet*(A), *dfrA1*, *aacA4*, *bla*_OXA-1_, *aadA2*
ICE*Sch*ST10	ST10	*Shewanella chilikensis*	*Sinonovacula constricta*	Nanjing city, Jiangsu Province, China, 2021.03	*qnrA1*, *tmexCD2-toprJ2*, *floR, strA*, *strB*, *tet*(A), *sul2*, *bla*_OXA-960_, *mph*(A), *sul1*, *bla*_NDM-1_	*tmexCD2-toprJ2*

### Transferability and fitness cost of SXT/R391 ICEs in tigecycline-resistant *Shewanella* isolates

A conjugation assay was performed to investigate the transferability of the three SXT/R391 ICE-harboring *Shewanella* isolates and the other 10 tigecycline-resistant *Shewanella* strains listed in Table S1. The *tet*(X4)-carrying ICEs exhibited a very low transfer frequency, ranging from 6.1 × 10^−7^ (strain NJT6) to 4.2 × 10^−7^ (strain NTT9) per recipient cell, indicating the possibility of *tet*(X4) gene dissemination via SXT/R391 ICEs. The transconjugants were also found to be resistant to antibiotics including chloramphenicol, tetracycline, and streptomycin (Table S1). However, *tmexCD2-toprJ2*-carrying ICEs (strain ST10) failed to transfer into *E. coli* C600 under test conditions. These results indicate that ICEs from different *Shewanella* strains show varying transferabilities. In addition, we found that the other tigecycline-resistant *Shewanella* strains, which did not harbor the *tet*(X) or *tmexCD-toprJ* gene, failed to transfer the tigecycline-resistant phenotype into *E. coli* C600 under test conditions. WGS revealed no tigecycline resistance genes in the genome sequences of these tigecycline-resistant strains. To investigate the potential role of efflux pumps in mediating tigecycline resistance, we assessed the impact of efflux pump inhibitors on the MICs of the eight non-transferable strains. Treatment with 1-(1-naphthylmethyl)-piperazine (NMP; 100 µg/mL) resulted in a remarkable 32-fold reduction in tigecycline MICs across all strains, while carbonyl cyanide m-chlorophenylhydrazone (100 µg/mL) treatment led to a more modest twofold decrease. These results strongly suggest that efflux pump activity plays a significant role in the observed tigecycline resistance. Notably, the pronounced effect of NMP—a known inhibitor of RND-type efflux pumps—particularly implicates this family of transporters in the resistance mechanism. To further elucidate the specific efflux systems involved, we are currently conducting RNA-seq analyses to identify differentially expressed efflux pump genes under tigecycline selection pressure (data not shown). Moreover, *E. coli* C600 showed a low competitive advantage over the transconjugants of NJT6 or NTT9 in the pairwise growth competition assay (Fig. S6). These results indicate that ICE*Sal*NJT6 and ICE*Sch*NTT9 exhibit undetectable fitness costs in host bacteria and could persist in bacteria of other species for a period of time, with the potential for further dissemination.

### Phylogenetic relationships between SXT/R391 ICEs and *Shewanella* isolates

**Fig 2:**
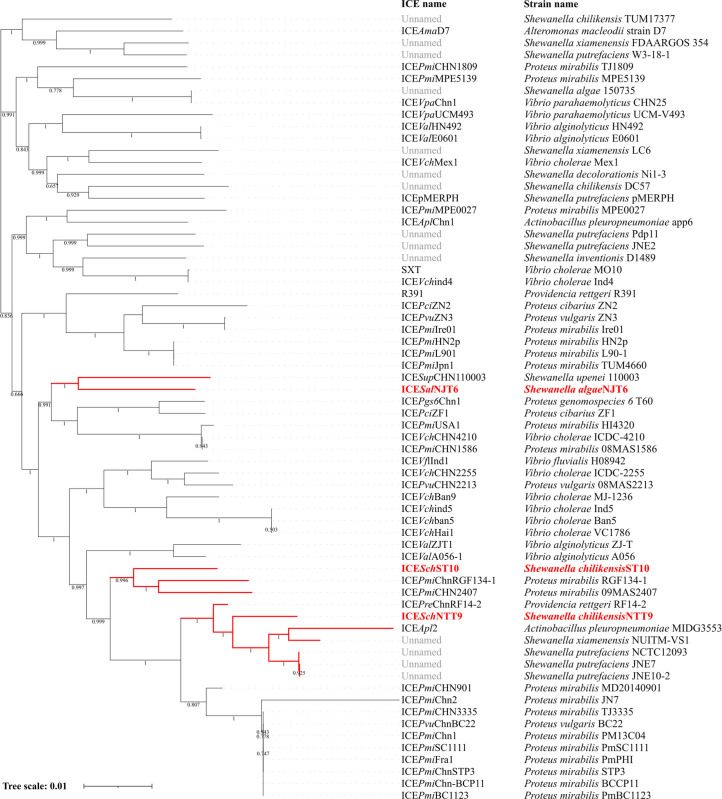
Phylogenetic analysis of the identified and representative SXT/R391 ICEs. The phylogenetic tree was constructed based on the SNPs in the conserved core genes of the ICEs via the maximum-likelihood method. ICEs identified in this study and the corresponding branches are highlighted in red.

To investigate the phylogenetic relationship of ICE*Sal*NJT6, ICE*Sch*NTT9, and ICE*Sch*ST10 with other known ICEs, phylogenetic analysis was performed (Fig. 2). ICE*Sal*NJT6 was classified into an independent cluster with ICE*Sup*CHN110003, present in *Shewanella upenei* strain 110003, which was isolated from a stool sample of a patient in Anhui province, China (37). Meanwhile, ICE*Sch*NTT9 was classified into a distinct branch adjacent to the cluster composed of ICE*Apl*2 carried by *Actinobacillus pleuropneumoniae* and uncharacterized ICEs in *S. putrefaciens* and *S. xiamenensis*. It is likely that both ICE*Sal*NJT6 and ICE*Sch*NTT9 harboring *tet*(X4) have a close evolutionary relationship with ICEs of other *Shewanella* species. However, ICE*Sch*ST10 harboring the *tmexCD2-toprJ2* gene formed a unique branch along with ICE*Pmi*ChnRGF134-1 and ICE*Pmi*CHN2407 carried by *Proteus mirabilis*. Notably, ICE*Pmi*ChnRGF134-1 also carried the *tmexCD-toprJ* gene cluster. This indicated that ICE*Sch*ST10 may share the same ancestor with these *Proteus* ICEs. To the best of our knowledge, this is the first report on the presence of *tet*(X4)- and *tmexCD2-toprJ2*-carrying SXT/R391 ICEs in *Shewanella* species. Altogether, our results indicate that ICE*Sal*NJT6, ICE*Sch*NTT9, and ICE*Sch*ST10 are novel members of the SXT/R391 family of ICEs and that SXT/R391 ICEs could be transferred horizontally in different bacterial species.

**Fig 3:**
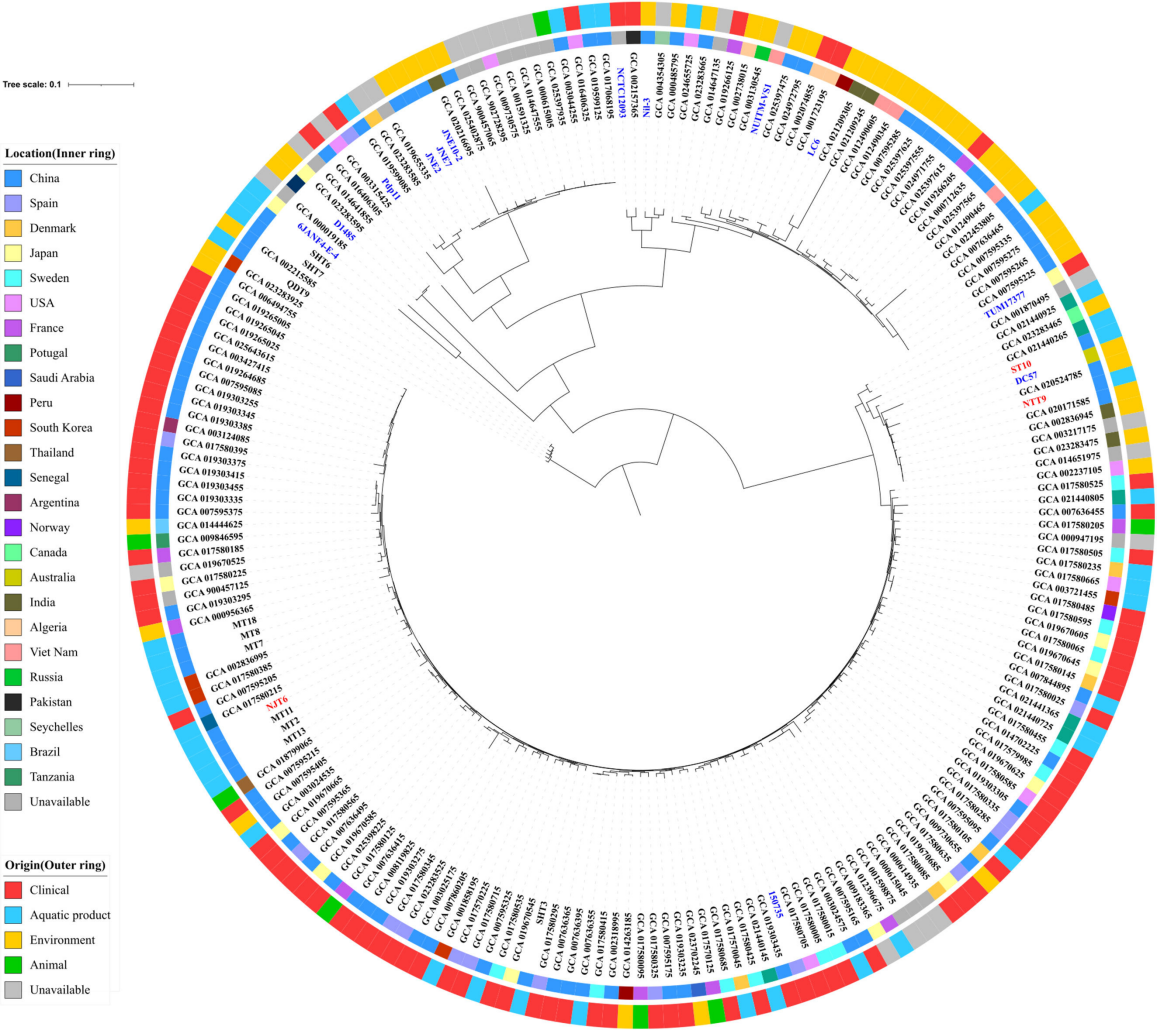
Phylogenetic analysis of the isolated and representative *Shewanella* strains. The phylogenetic tree was constructed based on the SNPs in the core genes of the *Shewanella* strains. Strains carrying ICEs in this study are highlighted in red, and other strains carrying ICEs are labeled in blue.

We retrieved genome sequences of *Shewanella* strains deposited in the NCBI database and found that several uncharacterized SXT/R391 ICEs were encoded by *S. algae*, *S. chilikensis*, *Shewanella decolorationis*, *Shewanella hafniensis*, *Shewanella inventionis*, and *S. xiamenensis*. Further bioinformatics analysis showed that ICEs are not distributed widely among *Shewanella* species. In addition, SNP analysis revealed that the strains NJT6, NTT9, and ST10 are not phylogenetically close to other *Shewanella* strains (Fig. 3). Since SXT/R391 ICE elements were also reported in *S. upenei* (37), *Shewanella halifaxensis* (21)*, S. putrefaciens* (38), and *Shewanella haliotis* (39), it is likely that *Shewanella* species are the reservoirs of SXT/R391 ICEs.

### Genomic context and variable regions in SXT/R391 ICEs

ICE*Sal*NJT6 was 104,326 bp long, with 47.2% guanine-cytosine (GC) content, while ICE*Sch*NTT9 was 112,127 bp in length, with 48.4% GC content. Additionally, ICE*Sal*NJT6 and ICE*Sch*NTT9 contained 86 and 111 predictive coding genes, respectively. BLASTn analysis showed that ICE*Sal*NJT6 exhibited high homologies with unnamed ICEs in *Proteus columbae* T60 and *S. putrefaciens* JNE7 with 96.53 and 96.21% identity, respectively, at 72% coverage (Fig. S1). Resistance genes *floR*, *strA*, *strB*, *sul2,* and insertion sequence IS*CR2* were also found in these two ICEs, but *tet*(X4) was not detected. Meanwhile, ICE*Sch*NTT9 exhibited the highest homology with an unnamed ICE in an *S. putrefaciens* strain JNE7, with 98.25% identity at 80% coverage (Fig. S2). In addition, ICE*Sch*ST10 carried by strain ST10 was 101,311 bp in length with 48.6% GC content and 96 predictive coding genes. Through BLASTn analysis, we found that ICE*Sch*ST10 was the most similar to ICE*Pmi*ChnRGF134-1 carried by the *P. mirabilis* strain RGF134-1 with 98.43% identity at 75% coverage (Fig. S3). The novel *tmexCD3-toprJ3* gene cluster and resistance genes, *sul2*, *strA*, *strB*, *floR*, were also found in ICE*Pmi*ChnRGF134-1. Notably, ICE*Pmi*ChnRGF134-1 also failed to transfer into *E. coli*. BLASTn against the NCBI database indicated that the *tmexCD-toprJ-*like gene cluster harbored by ICE*Sch*ST10 shared 99.83% nucleotide sequence identity with *tnfxB2-tmexC2D2.2-toprJ2* reported in *Klebsiella oxytoca* (40), indicating that ICE*Sch*ST10 carries the *tmexCD2-toprJ2* gene cluster.

**Fig 4:**
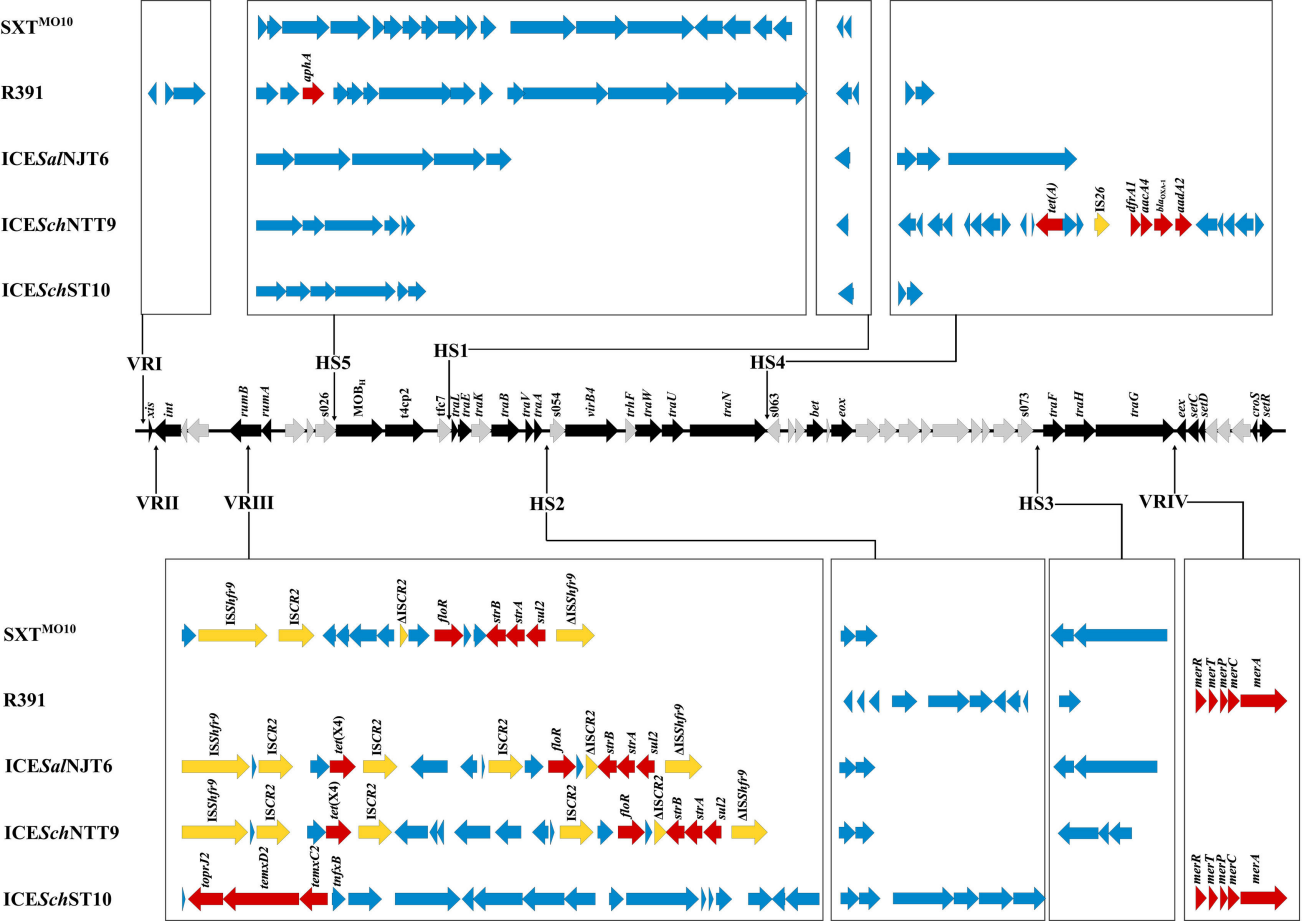
Genomic composition of the ICEs identified in this study. The middle line shows the backbone of the SXT/R391 ICEs with conserved core genes, and the black arrows represent the core genes used for the construction of the phylogenetic tree. The red and yellow arrows represent the drug resistance genes and insertion sequences, respectively. HS1–5 represent five hotspots, and VRI–IV represent four variable regions.

The backbones of ICE*Sch*NTT9 and ICE*Sch*ST10 were highly conserved, and both of them exhibited 96% similarity with the core backbone of the reference ICE SXT^MO10^. A total of HS1–5 and VRI–IV have been identified in these ICEs (Fig. 4). Notably, the VRIII in ICE*Sal*NJT6 and ICE*Sch*ST10 contained the same antibiotic resistance genes, including *strA*, *strB*, *sul*2, *floR*, *sul2*, and *tet*(X4). The *tet*(X4) gene was flanked by IS*CR2*, forming the IS*CR2-aph-tet*(X4)-IS*CR2* region, indicating that IS*CR2* may mediate the insertion of *tet*(X4) into these two ICEs (Fig. S4). In addition, ICE*Sch*ST10 carried an MDR region that was integrated into HS4, while the other four HS4 of ICEs did not carry any drug resistance genes. The resistance genes in the HS4 of ICE*Sch*ST10 contained *tet*(A), *dfrA1*, *aacA4*, *aadA2*, *bla*_OXA-1_, and *czcD* genes. Through BLASTn analysis, we found that a similar MDR region (query coverage >90%) was widely distributed in *P. mirabilis* strains. These resistance genes in the HS4 of ICE*Sch*ST10 were flanked by two copies of IS*Ppu12*, indicating that IS*Ppu12* may mediate the insertion of this MDR region into ICE*Sch*ST10. Further analysis showed that a region of 17,365 bp containing *tmexCD2-toprJ2* was inserted into the VRIII of ICE*Sch*ST10 (Fig. 4). Although the conjugation assay of ICE*Sch*ST10 failed under test conditions, the circular formation of ICE*Sch*ST10 was confirmed using two pairs of primers P1P2 and P3P4 listed in Table S4, indicating that the circular form of ICE*Sch*ST10 carrying *tmexC2D2-toprJ2* could be excised from the chromosome as the archetypical transfer process of ICE (Fig. S7).

Notably, *bla*_NDM-1_ was identified in strain ST10. We found that the *bla*_NDM-1_ gene was located in a genomic island, which was designated as SGI-ScNDM-1. SGI-ScNDM-1 was 87,737 bp in length with 52.7% GC content and 91 predictive coding genes. BLASTn analysis showed that the backbone of SGI-ScNDM-1 had high similarities with SGI1 members of other bacterial species. However, the variable region of SGI-ScNDM-1 was different from the known sequences in the NCBI database and contained *tet*(A), *sul1*, *mph(A*), and *bla*_NDM-1_ (Fig. S5). Several insertion sequences were also found in this variable region, such as IS*5075*, IS*Kpn26,* and IS*1326*. However, the functions of other genes encoded in this region were unknown.

### Conclusion

In conclusion, this is the first study to report on the emergence of novel SXT/R391 ICEs carrying *tet*(X4) and *tmexCD2-toprJ2* genes in *Shewanella* species of seafood origin. The identified SXT/R391 ICEs could mediate the dissemination of tigecycline resistance genes among aquatic bacteria. Tigecycline-resistant bacterial species are prevalent in retail seafood, and *Shewanella* spp. are likely the potential reservoirs of tigecycline-resistance genes in aquatic products. Contact between seafood and consumers may lead to the dissemination of tigecycline-resistance genes in humans. Therefore, it is imperative to increase our awareness of the emergence of tigecycline-resistant aquatic bacteria.

## Data Availability

The complete sequences of ST10 have been deposited in the NCBI database with accession number CP149966. The genome sequences of NJT6, NTT9, and other 10 *Shewanella* strains were involved in the BioProject PRJNA1227024.

## SUPPLEMENTAL MATERIAL

Supplemental material aac.01742-24-s0001.docx Tables S1 to S5; Fig. S1 to S7.

ASM does not own the copyrights to Supplemental Material that may be linked to, or accessed through, an article. The authors have granted ASM a non-exclusive, world-wide license to publish the Supplemental Material files. Please contact the corresponding author directly for reuse.
