# Supplementary material for: Detection of SXT/R391 integrative conjugative elements carrying tigecycline resistance genes in Shewanella spp. isolated from retail seafood
Source: Antimicrob Agents Chemother. 2025 Jun 26;69(8):e01742-24. doi: 10.1128/aac.01742-24 (PMC12326977; doi:10.1128/aac.01742-24)
Supplement: Supplemental material — Tables S1 to S5; Fig. S1 to S7. [file aac.01742-24-s0001.docx]

**Supplementary Data**

**Table S1. MICs of the *Shewanella* isolates and transconjugants in this study.**

| **Strain** | **Species** | **MIC** | | | | | | | | | | | | | | |
| --- | --- | --- | --- | --- | --- | --- | --- | --- | --- | --- | --- | --- | --- | --- | --- | --- |
|  |  | **MER** | **IMP** | **TIG** | **COL** | **CFF** | **CIP** | **CHL** | **TET** | **KAN** | **GEN** | **STR** | **CRO** | **ATM** | **AMK** | **AMP** |
| NJT6 | *Shewanella algae* | ≤0.25 | 2 | 32 | 0.5 | ＞128 | 2 | 64 | ＞128 | 16 | 1 | ＞128 | 0.5 | ＞128 | 1 | 2 |
| JC600-NJT6 | *E. coli* | ≤0.25 | ≤0.25 | 16 | ≤0.25 | 1 | ≤0.25 | ＞128 | ＞128 | 2 | 0.5 | ＞128 | ≤0.25 | 1 | 1 | 16 |
| NTT9 | *Shewanella chilikensis* | ≤0.25 | 1 | 32 | 128 | 32 | 32 | 64 | 64 | ＞128 | 8 | ＞128 | 0.5 | ＞128 | 2 | ＞128 |
| JC600-NTT9 | *E. coli* | ≤0.25 | ≤0.25 | 16 | ≤0.25 | 1 | ≤0.25 | 64 | 64 | ＞128 | 4 | ＞128 | ≤0.25 | 0.5 | 2 | ＞128 |
| ST10 | *Shewanella chilikensis* | ＞128 | ＞128 | 8 | 4 | ＞128 | 16 | ＞128 | 32 | ＞128 | 0.5 | ＞128 | ＞128 | ≤0.25 | 2 | ＞128 |
| MT13 | *Shewanella algae* | ≤0.25 | 4 | 4 | ＞128 | 64 | ＞128 | 64 | 8 | 2 | 1 | ＞128 | 64 | ＞128 | 1 | ＞128 |
| QDT9 | *Shewanella marisflavi* | ≤0.25 | 1 | 4 | ≤0.25 | 1 | 4 | 128 | 16 | 4 | 1 | ＞128 | ≤0.25 | ≤0.25 | 1 | ＞128 |
| SHT6 | *Shewanella marisflavi* | ≤0.25 | 1 | 4 | ≤0.25 | 2 | 64 | 128 | 16 | 2 | 0.5 | ＞128 | ≤0.25 | ≤0.25 | 1 | ＞128 |
| SHT7 | *Shewanella marisflavi* | ≤0.25 | 1 | 4 | ≤0.25 | 2 | 64 | 128 | 16 | 2 | 0.5 | ＞128 | ≤0.25 | ≤0.25 | 1 | ＞128 |
| SHT3 | *Shewanella algae* | ≤0.25 | 4 | 8 | ≤0.25 | 0.5 | ＞128 | ＞128 | 16 | ＞128 | 8 | ＞128 | ≤0.25 | ≤0.25 | 2 | ＞128 |
| MT8 | *Shewanella algae* | ≤0.25 | 2 | 8 | ≤0.25 | 2 | ＞128 | 128 | 32 | 4 | 1 | 16 | 64 | 32 | 1 | ＞128 |
| MT7 | *Shewanella algae* | ≤0.25 | 2 | 4 | ≤0.25 | 32 | 128 | 128 | 16 | 4 | 0.5 | ＞128 | 32 | ＞128 | 2 | ＞128 |
| MT11 | *Shewanella algae* | ≤0.25 | 4 | 4 | ＞128 | 64 | ＞128 | 64 | 8 | 2 | 1 | ＞128 | 64 | ＞128 | 1 | ＞128 |
| MT2 | *Shewanella algae* | ≤0.25 | 4 | 4 | ＞128 | 32 | 128 | 64 | 16 | 4 | 1 | ＞128 | 32 | ＞128 | 1 | ＞128 |
| MT18 | *Shewanella algae* | ≤0.25 | 2 | 8 | ≤0.25 | 2 | ＞128 | 128 | 32 | 4 | 1 | 16 | 64 | 32 | 1 | ＞128 |

MER, meropenem; IMP, imipenem; TIG, tigecycline; COL, colistin; CFF, ceftiofur; CIP, ciprofloxacin; CHL, chloramphenicol; TET, tetracycline; KAN, kanamycin; GEN, gentemycin; STR, streptomycin; CRO, ceftriaxone; ATM, aztreonam; AMK, amikacin; AMP, ampicillin

**Table S2. Characteristics of the representative *Shewanella* strains used for phylogenetic analysis**

| Code | Strain | Location | Origin | Collection date |
| --- | --- | --- | --- | --- |
| GCA_017580285 | 6F5 | Spain | Clinical sample | 2011 |
| GCA_007595095 | SYT1 | China | Aquatic products | 2014 |
| GCA_019303305 | LZ201228 | China | Clinical sample | 2012 |
| GCA_017580335 | 590722 | Spain | Clinical sample | 2018 |
| GCA_017580105 | A94 | Denmark | Clinical sample | 1994 |
| GCA_009730655 | RQs-106 | China | Environment | 2011 |
| GCA_017580635 | 5043 | Spain | Clinical sample | 2015 |
| GCA_019670685 | TUM17386 | Japan | Clinical sample | 2015 |
| GCA_017580085 | A97 | Denmark | Clinical sample | 1992 |
| GCA_017580455 | CCUG-56496 | Sweden | Clinical sample | 2008 |
| GCA_014702225 | VGH117 | China | Clinical sample | 2015 |
| GCA_017579985 | HUD-D8 | Sweden | Clinical sample | 2016 |
| GCA_019670625 | TUM17382 | Japan | Clinical sample | 2015 |
| GCA_017580585 | CCUG-15259 | USA | Clinical sample | 1984 |
| GCA_000614935 | JCM 14758 | Unavailable | Unavailable | Unavailable |
| GCA_000615045 | JCM 21037 | Unavailable | Unavailable | Unavailable |
| GCA_001598875 | NBRC 103173 | Unavailable | Unavailable | Unavailable |
| GCA_012396675 | ATCC 51192 | France | Aquatic products | Unavailable |
| GCA_009183365 | CECT-5071 | Japan | Unavailable | 1990 |
| GCA_017580015 | HUD-H4 | Sweden | Clinical sample | 2018 |
| GCA_017580005 | HUD-I2 | Sweden | Clinical sample | 2018 |
| GCA_007595165 | CLS4 | China | Clinical sample | 2014 |
| GCA_003024575 | SYT4 | China | Aquatic products | 2014 |
| GCA_017580705 | CCUG-789 | USA | Clinical sample | 1970 |
| GCA_017570325 | 150735 | Spain | Clinical sample | 2015 |
| GCA_019303435 | 08MAS2314 | China | Clinical sample | 2008 |
| GCA_021440145 | INSAq494 | Potugal | Aquatic products | 2019 |
| GCA_017580425 | CCUG-72638 | Sweden | Clinical sample | 2018 |
| GCA_017570045 | A291 | Denmark | Aquatic products | 1995 |
| GCA_017580685 | CCUG-526 | Sweden | Clinical sample | 1969 |
| GCA_017570125 | A59 | France | wild animal | 1980 |
| GCA_023702245 | B29 | Saudi Arabia | Environment | 2022 |
| GCA_019303235 | LZ2015243 | China | Clinical sample | 2015 |
| GCA_007595175 | CLS5 | China | Clinical sample | 2014 |
| GCA_017580325 | 669801 | Spain | Clinical sample | 2016 |
| GCA_017580095 | A60 | France | wild animal | 1980 |
| GCA_014263185 | 2NE11 | Peru | Environment | 2018 |
| GCA_002318995 | YHL | China | Clinical sample | 2014 |
| GCA_017580415 | CCUG-72678 | Sweden | Clinical sample | 2018 |
| GCA_007636355 | JFC3 | China | Aquatic products | 2014 |
| GCA_007636395 | RC | China | Clinical sample | 2014 |
| GCA_007636365 | YTH | China | Clinical sample | 2014 |
| GCA_017580295 | 950570 | Spain | Clinical sample | 2019 |
| GCA_017580535 | CCUG-50501 | Sweden | Clinical sample | 2010 |
| GCA_007595325 | SYT2 | China | Aquatic products | 2014 |
| SHT3 | SHT3 | China | Aquatic products | 2021 |
| GCA_019670545 | TUM4442 | Japan | Clinical sample | Unavailable |
| GCA_017580715 | 404 | Spain | Clinical sample | 2008 |
| GCA_017570225 | G1 | Spain | Clinical sample | 2016 |
| GCA_001858195 | CSB04KR | South Korea | Aquatic products | 2015 |
| GCA_007860205 | JFC1 | China | Clinical sample | 2014 |
| GCA_003025175 | ACCC | China | Clinical sample | 2014 |
| GCA_023283525 | 254-1 | Spain | Clinical sample | 2014 |
| GCA_017580345 | 159418 | Spain | Clinical sample | 2015 |
| GCA_019303275 | LZ2015256 | China | Clinical sample | 2015 |
| GCA_008119825 | SYC | China | Clinical sample | 2014 |
| GCA_007636415 | YTL | China | Clinical sample | 2014 |
| GCA_017580125 | A65 | France | poultry | 1982 |
| GCA_025398225 | HN_2022 | China | Clinical sample | 2022 |
| GCA_019670585 | TUM17378 | Japan | Clinical sample | 2014 |
| GCA_007636495 | TYL | China | Clinical sample | 2014 |
| GCA_017580565 | 28011 | Spain | Clinical sample | 2014 |
| GCA_007595365 | CLS2 | China | Clinical sample | 2014 |
| GCA_018799065 | Colony281 | Thailand | Food | Unavailable |
| MT13 | MT13 | China | Aquatic products | 2021 |
| MT2 | MT2 | China | Aquatic products | 2021 |
| MT11 | MT11 | China | Aquatic products | 2021 |
| GCA_019670665 | TUM17384 | Japan | Clinical sample | 2015 |
| GCA_003024535 | AC | China | Aquatic products | 2014 |
| GCA_007595405 | SYT3 | China | Environment | 2014 |
| GCA_007595215 | melkephyllucas | China | Clinical sample | 2014 |
| NJT6 | NJT6 | China | Aquatic products | 2022 |
| GCA_017580215 | A56 | Senegal | Aquatic products | 1980 |
| GCA_007595205 | CHL | China | Clinical sample | 2014 |
| GCA_017580385 | CCUG-58400 | South Korea | Aquatic products | 2009 |
| GCA_002836995 | 20-23R | South Korea | Aquatic products | Unavailable |
| MT7 | MT7 | China | Aquatic products | 2021 |
| MT8 | MT8 | China | Aquatic products | 2021 |
| MT18 | MT18 | China | Aquatic products | 2021 |
| GCA_000956365 | C6G3 | France | Environment | 2007 |
| GCA_019303295 | MAS2736 | China | Clinical sample | 2007 |
| GCA_900457125 | NCTC10738 | Unavailable | Clinical sample | 1970 |
| GCA_017580225 | A41 | Japan | Clinical sample | 1963 |
| GCA_019670525 | ATCC49138 | Unavailable | Unavailable | Unavailable |
| GCA_017580185 | A57 | France | Clinical sample | 1980 |
| GCA_009846595 | 18064-CSB-B-B | Tanzania | poultry | 2018 |
| GCA_014444625 | A3/19 | Brazil | Environment | 2019 |
| GCA_007595375 | CLS3 | China | Clinical sample | 2014 |
| GCA_019303415 | LC2016-2 | China | Clinical sample | 2016 |
| GCA_019303375 | LC2016-3 | China | Clinical sample | 2016 |
| GCA_019303335 | LC2016-4 | China | Clinical sample | 2016 |
| GCA_019303455 | LC2016-1 | China | Clinical sample | 2016 |
| GCA_017580395 | 97087 | Spain | Clinical sample | 2015 |
| GCA_003124085 | Sh392 | Argentina | Clinical sample | 2006 |
| GCA_019303385 | LC2016-6 | China | Clinical sample | 2016 |
| GCA_019303345 | LC2016-5 | China | Clinical sample | 2016 |
| GCA_019303255 | LZ2013652 | China | Clinical sample | 2013 |
| GCA_007595085 | CLS1 | China | Clinical sample | 2014 |
| GCA_019264685 | CCU4051 | China | Clinical sample | 2016 |
| GCA_003427415 | CCU101 | China | Clinical sample | 2016 |
| GCA_019265045 | CCU4054 | China | Clinical sample | 2016 |
| GCA_019265005 | CCU4052 | China | Clinical sample | 2016 |
| GCA_025643615 | VGH117 | China | Clinical sample | 2015 |
| GCA_019265025 | CCU4053 | China | Clinical sample | 2016 |
| GCA_017580025 | SF7 | Spain | Clinical sample | 2010 |
| GCA_021441365 | INSAq258 | Potugal | Aquatic products | 2019 |
| GCA_021440725 | INSAq495 | Potugal | Aquatic products | 2019 |
| GCA_019670645 | TUM17383 | Japan | Clinical sample | 2015 |
| GCA_017580145 | A93 | Denmark | Clinical sample | 1994 |
| GCA_007844895 | JFC2 | China | Aquatic products | 2014 |
| GCA_000947195 | MARS 14 | Unavailable | Unavailable | 2015 |
| GCA_017580505 | CCUG-48086 | Sweden | Clinical sample | 2003 |
| GCA_017580235 | A292 | Denmark | Aquatic products | 1995 |
| GCA_017580665 | CCUG-12945 | USA | Aquatic products | 1982 |
| GCA_003721455 | KC-Na-R1 | South Korea | Aquatic products | 2017 |
| GCA_017580485 | CCUG-38646 | Norway | Clinical sample | 1997 |
| GCA_017580595 | CCUG-20533 | Sweden | Clinical sample | 1987 |
| GCA_019670605 | TUM17379 | Japan | Clinical sample | 2014 |
| GCA_017580065 | HUD-G3 | Sweden | Clinical sample | 2018 |
| GCA_017580205 | A58 | France | wild animal | 1980 |
| GCA_021440805 | INSAq497 | Potugal | Aquatic products | 2019 |
| GCA_007636455 | JFL | China | Clinical sample | 2014 |
| GCA_017580525 | CCUG-24987 | Sweden | Clinical sample | 1989 |
| GCA_002237105 | MN-01 | USA | Environment | 2010 |
| GCA_019670565 | TUM17377 | Japan | Clinical sample | 2012 |
| GCA_001870495 | BrY | Unavailable | Unavailable | Unavailable |
| GCA_021440925 | INSAq334 | Potugal | Aquatic products | 2019 |
| GCA_023283465 | A49 | Canada | Environment | 1987 |
| GCA_021440265 | INSAq243 | Potugal | Aquatic products | 2019 |
| ST10 | ST10 | China | Aquatic products | 2021 |
| GCA_011106835 | DC57 | Australia | Environment | 2016 |
| GCA_020524785 | SaN7-13 | China | Environment | 2021 |
| NTT9 | NTT9 | China | Aquatic products | 2022 |
| GCA_020171585 | AmN21-7 | China | Environment | 2021 |
| GCA_002836945 | JC5 | India | Environment | 2007 |
| GCA_003217175 | JC5 | Unavailable | Unavailable | Unavailable |
| GCA_023283475 | CCUG 57101 | India | Environment | 2007 |
| GCA_014651975 | KCTC 22540 | Unavailable | Unavailable | Unavailable |
| GCA_006494755 | ECSMB14101 | China | Environment | 2014 |
| GCA_023283925 | JCM 12192 | South Korea | Environment | 2004 |
| QDT9 | QDT9 | China | Aquatic products | 2021 |
| GCA_002215585 | EP1 | China | Environment | 2007 |
| SHT7 | SHT7 | China | Aquatic products | 2022 |
| SHT6 | SHT7 | China | Aquatic products | 2022 |
| GCA_003112715 | 6JANF4-E-4 | Japan | Aquatic products | 2013 |
| GCA_000019185 | HAW-EB4 | Unavailable | Unavailable | Unavailable |
| GCA_019931735 | D1489 | Pacific Ocean | Environment | 2017 |
| GCA_023283595 | KCTC 42807 | Japan | Environment | 2014 |
| GCA_014641855 | CGMCC 1.15339 | Unavailable | Unavailable | Unavailable |
| GCA_016406305 | CGMCC-1.6515 | China | Clinical sample | 2018 |
| GCA_003315425 | 97 | USA | Unavailable | Unavailable |
| GCA_023283585 | KCTC 22180 | Denmark | Unavailable | 2001 |
| GCA_019655335 | P010 | Unavailable | Unavailable | Unavailable |
| ShewanellaPdp11 | Pdp11 | Spain | Clinical sample | 2005 |
| GCA_019599085 | YZ08 | China | Aquatic products | 2021 |
| GCA_023159115 | JNE2 | China | Environment | 2021 |
| GCA_023159135 | JNE7 | China | Environment | 2021 |
| GCA_023159095 | JNE10-2 | China | Environment | 2021 |
| GCA_020216695 | M101 | India | Environment | 2017 |
| GCA_025402875 | 4H | China | Environment | 2015 |
| GCA_900457065 | NCTC:10695 | Unavailable | Unavailable | Unavailable |
| GCA_902728295 | T2.3D-1.1 | Unavailable | Unavailable | 2020 |
| GCA_009730575 | FDAARGOS_681 | USA | Unavailable | 1994 |
| GCA_001591325 | NBRC 3908 | Unavailable | Unavailable | Unavailable |
| GCA_014647555 | JCM 20190 | Unavailable | Unavailable | Unavailable |
| GCA_000615005 | DRS002684 | Unavailable | Unavailable | Unavailable |
| GCA_025397935 | DSM 50426 | Unavailable | Food | 1990 |
| GCA_003044255 | WS13 | China | Aquatic products | 2014 |
| GCA_016406325 | ATCC 8071 | USA | Clinical sample | 2018 |
| GCA_019599125 | YZ-J | China | Aquatic products | 2021 |
| GCA_017068195 | XY07 | China | Aquatic products | 2018 |
| GCA_900636665 | NCTC12093 | Unavailable | Clinical sample | 1987 |
| GCA_002157365 | SA70 | Pakistan | Clinical sample | 2016 |
| GCA_007923045 | Ni1-3 | China | Environment | 2017 |
| GCA_004354305 | sesselensis | Seychelles | Unavailable | 2014 |
| GCA_000485795 | S12 | China | Environment | 2012 |
| GCA_024655725 | 28.1.37 | USA | Aquatic products | 2022 |
| GCA_023283665 | DSM 22215 | China | Environment | 2010 |
| GCA_014647135 | JCM 16212 | Unavailable | Unavailable | Unavailable |
| GCA_019266125 | 111D | France | Clinical sample | 2013 |
| GCA_002738015 | AS58 | Algeria | Environment | 2015 |
| GCA_003130545 | DCB2-1 | Russia | Environment | 2017 |
| GCA_019973655 | NUITM-VS1 | Viet Nam | Unavailable | 2021 |
| GCA_025397475 | HD6449 | China | Environment | 2019 |
| GCA_024972795 | HD6452 | China | Environment | 2019 |
| GCA_002074855 | T17 | Algeria | Clinical sample | 2012 |
| GCA_001723195 | T17 | Algeria | Clinical sample | 2012 |
| Shewanella_LC6 | LC6 | Peru | Environment | 2016 |
| GCA_021209305 | S2C505 | India | Environment | 2018 |
| GCA_021209245 | S2C505 | India | Environment | 2018 |
| GCA_012490605 | SxND_W9_2018 | Viet Nam | Environment | 2018 |
| GCA_012490345 | SxND_W9_2018 | Viet Nam | Environment | 2018 |
| GCA_007595285 | ZYW6 | China | Environment | 2014 |
| GCA_025397625 | HD6420 | China | Environment | 2019 |
| GCA_025397555 | HD6446 | China | Environment | 2019 |
| GCA_024971755 | HD6416 | China | Environment | 2019 |
| GCA_025397615 | HD6424 | China | Environment | 2019 |
| GCA_019266205 | 111B | France | Clinical sample | 2013 |
| GCA_000712635 | BC01 | China | Environment | 2011 |
| GCA_025397565 | HD6443 | China | Environment | 2019 |
| GCA_012490465 | SxND_W5_2018 | Viet Nam | Environment | 2018 |
| GCA_022453805 | CQ-Y1 | China | Environment | 2020 |
| GCA_007595225 | ZYW2 | China | Environment | 2014 |
| GCA_007595265 | ZYW3 | China | Environment | 2014 |
| GCA_007595275 | ZYW4 | China | Environment | 2014 |
| GCA_007595335 | ZYW5 | China | Environment | 2014 |
| GCA_007636465 | ZYW2 | China | Aquatic products | 2014 |

**Table S3. Characteristics of the representative ICEs used for phylogenetic analysis.** NA, information not available

| **ICEs** | **Strain** | **Source** | **Location** | **Year** | **Accession number** |
| --- | --- | --- | --- | --- | --- |
| SXT^MO10^ | *Vibrio cholerae* MO10 | Clinical sample | Chennai, India | 2002 | AY055428 |
| R391 | *Providencia rettgeri* R391 | Stool | Pretoria, South Africa | 1967 | AY090559 |
| ICE*Sup*CHN110003 | *Shewanella upenei* 110003 | Clinical sample | Anhui, China | 2011 | MG014393 |
| ICE*Spu*PO1 | *Shewanella putrefaciens* W3-18-1 | Marine sediments | Pacific Ocean | 2000 | CP000503 |
| ICEpMERPH | *Shewanella putrefaciens* pMERPH | River mersey | UK | 1987 | MH974755 |
| NA | *Shewanella inventionis* D1489 | Cerebrospinal fluid | USA | 1987 | CP082926 |
| NA | *Shewanella putrefaciens* JNE2 | Environment | Jiangsu, China | 2021 | CP096169 |
| NA | *Shewanella hafniensis* Pdp11 | Sparus aurata | Spain | 2005 | CP015194 |
| NA | *Shewanella xiamenensis* NUITM-VS1 | NA | Viet Nam | 2021 | AP025014 |
| NA | *Shewanella putrefaciens* NCTC12093 | Cerebrospinal fluid | USA | 1987 | LR134303 |
| NA | *Shewanella putrefaciens* JNE7 | Environment | Jiangsu, China | 2021 | CP096166 |
| NA | *Shewanella putrefaciens* JNE10-2 | Environment | Jiangsu, China | 2021 | CP096163 |
| NA | *Shewanella algae*  150735 | Human wound | Spain | 2015 | CP068229 |
| NA | *Shewanella decolorationis* Ni1-3 | Environment | China | 2017 | CP031775 |
| NA | *Shewanella chilikensis* TUM17377 | Human wound | Japan | 2012 | AP024611 |
| NA | *Shewanella xiamenensis.* LC6 | Environment | Peru | 2016 | CP043902 |
| NA | *Shewanella xiamenensis.* FDAARGOS_354 | Environment | USA | 2016 | CP022089 |
| NA | *Shewanella chilikensis* DC57 | Corroded pipe | Australia | 2016 | CP045857 |
| ICE*Vfl*Ind1 | *Vibrio fluvialis* H08942 | Diarrhea | Kolkata, India | 2002 | KM213605 |
| ICE*Vch*Ind5 | *Vibrio cholerae* Ind5 | NA | Sevagram, India | 1994 | GQ463142 |
| ICE*Vch*Ind4 | *Vibrio cholerae* Ind4 | Clinical | Kolkata, India | 1997 | GQ463141 |
| ICE*Vch*CHN2255 | *Vibrio cholerae* ICDC-2255 | Patient | Hainan, China | 2008 | KT151660 |
| ICE*Vch*Hai1 | *Vibrio cholerae* VC1786 | Stool | Artibonite, Haiti | 2010 | JN648379 |
| ICE*Vch*Ban5 | *Vibrio cholerae* Ban5 | NA | Bangladesh | 1998 | GQ463140 |
| ICE*Vch*Ban9 | *Vibrio cholerae* MJ-1236 | NA | Bangladesh | 1994 | CP001485 |
| ICE*Vch*CHN4210 | *Vibrio cholerae* ICDC-4210 | Patient | Jiangxi, China | 1999 | KT151662 |
| ICE*Vch*Mex1 | *Vibrio cholerae* Mex1 | Sewage | San Luis Potosi, Mexico | 2001 | GQ463143 |
| ICE*Vpa*UCM493 | *Vibrio parahaemolyticus* UCM-V493 | Sediment | Spain | 2002 | CP007004 |
| ICE*Val*ZJT1 | *Vibrio alginolyticus* ZJ-T | Orange-spotted grouper | Guangdong, China | 2005 | CP016224 |
| ICE*Val*HN492 | *Vibrio alginolyticus* HN492 | Seawater | Guangdong, China | 2008 | KT072769 |
| ICE*Val*E0601 | *Vibrio alginolyticus* E0601 | Seawater | Guangdong, China | 2006 | KT072768 |
| ICE*Val*A056-1 | *Vibrio alginolyticus A*056 | White leg shrimp | Guangdong, China | 2003 | KR231688 |
| ICEVpaChn1 | Vibrio parahaemolyticus CHN25 | Shrimp | Shanghai, China | 2011 | CP010883 |
| ICE*Pmi*Jpn1 | *Proteus mirabilis*TUM4660 | Clinical sample | Japan | 2006 | KY437729 |
| ICE*Pmi*ChnRGF134-1 | *Proteus mirabilis* RGF134-1 | Pig | Jiangsu,  China | 2019 | CP066833 |
| ICE*Pmi*ChnSTP3 | *Proteus mirabilis* STP3 | Pig | Sichuan, China | 2019 | MT449450 |
| ICE*Pmi*Chn1 | *Proteus mirabilis* PM13C04 | Chicken | Hubei, China | 2013 | KT962845 |
| ICE*Pmi*Chn-BCP11 | *Proteus mirabilis* BCCP11 | Pig | Sichuan, China | 2016 | MG773277 |
| ICE*Pmi*CHN3335 | *Proteus mirabilis* TJ3335 | Stool | Tianjin, China | 2013 | KX243416 |
| ICE*Pmi*Chn2 | *Proteus mirabilis* JN7 | Broiler carcasses | Shandong, China | 2013 | KY437726 |
| ICE*Pmi*SC1111 | *Proteus mirabilis* PmSC1111 | Swine | Mianyang, China | 2017 | CP034090 |
| ICE*Pmi*MPE5139 | *Proteus mirabilis* MPE5139 | Feces | Guangzhou, China | 2019 | CP053684 |
| ICE*Pmi*MPE0027 | *Proteus mirabilis* MPE0027 | Feces | Shanghai, China | 2018 | CP053683 |
| ICE*Pmi*BC1123 | *Proteus mirabilis* PmBC1123 | Swine | Mianyang, China | 2017 | CP034091 |
| ICE*Pmi*HN2p | *Proteus mirabilis* HN2p | Swine | Henan, China | 2019 | CP046048 |
| ICE*Pmi*L901 | *Proteus mirabilis* L90-1 | Stool | Hangzhou, China | 2016 | CP045257 |
| ICE*Pmi*Fra1 | *Proteus mirabilis* PmPHI | Clinical sample | France | 2012 | MF490434 |
| ICE*Pm*iCHN901 | *Proteus mirabilis* MD20140901 | Stool | Beijing, China | 2014 | KX243408 |
| ICE*Pmi*CHN2407 | *Proteus mirabilis* 09MAS2407 | Stool | Maanshan, China | 2008 | KX243405 |
| ICE*Pmi*CHN1586 | *Proteus mirabilis* 08MAS1586 | Food | Maanshan, China | 2008 | KX243404 |
| ICE*Pmi*CHN1809 | *Proteus mirabilis T*J1809 | Stool | Tianjin, China | 2013 | KX243413 |
| ICE*Pmi*USA1 | *Proteus mirabilis* HI4320 | Human urine | Maryland, USA | 1986 | AM942759 |
| ICE*Pmi*Ire01 | *Proteus mirabilis* Ire01 | Wastewater | Dublin, Ireland | 2018 | MN520463 |
| ICE*Pci*ZF1 | *Proteus cibarius* ZF1 | Swine feces | Nantong, China | 2018 | CP047340 |
| ICE*Pci*ZN2 | *Proteus cibarius* ZN2 | Swine nose swab sample | Nantong, China | 2018 | CP047349 |
| ICE*Pvu*ZN3 | *Proteus vulgaris* ZN3 | Swine nose swab | Nantong, China | 2018 | CP047344 |
| ICE*Pvu*CHN2213 | *Proteus vulgaris* 08MAS2213 | Food | Maansham, China | 2008 | KX243403 |
| ICEPvuChnBC22 | *Proteus vulgaris* BC22 | Swine anal swab | China | 2018 | MH160822 |
| ICE*Pgs6*Chn1 | *Proteus genomospecies 6* T60 | Retail  meat | Henan, China | 2019 | MN507533 |
| ICE*Pre*ChnRF14-2 | *Providencia rettgeri* RF14-2 | Pig | Jiangsu, China | 2020 | MT219827 |
| ICE*Apl*2 | *Actinobacillus pleuropneumoniae* MIDG3553 | Pig | UK | 2012 | MF187965 |
| ICE*Apl*Chn1 | *Actinobacillus pleuropneumoniae* app6 | Pig | Shanghai, China | 2013 | KX196444 |
| ICE*Ama*D7 | *Alteromonas macleodii* strain D7 | Seawater | Andaman Sea, Thailand | 2000 | CP014323 |
| ICE*Ame*UM7 | *Alteromonas editerrânea* UM7 | Water | Ionian Sea | NA | CP004853 |

**Table S4. Primer sequences used for PCR amplification.**

| **Primers** | **Primer Sequence（5’-3’）** | **Targets** |
| --- | --- | --- |
| *tet*(X)-F | CCGATATTCATCATCCAGAGG | *tet*(X) gene |
| *tet*(X)-R | CGCTTACTTTTCCAAGACTTACCT |  |
| *tmexCD*-F | CAGCCAGGACTACAACTTC | *tmexCD* gene cluster |
| *tmexCD*-R | TAGAGGAACTTCGGATTGC |  |
| P1 | ACGGAAGAACATGTCAGGTA | *prfC* after excision of ICE*Sch*ST10 |
| P2 | CCTGGATCTTGAACACAAA |  |
| P3 | TGCTGTCATCTGCATTCTCCTG | Circular form of ICE*Sch*ST10 |
| P4 | GCCAATTACGATTAACACGACGG |  |

**Table S5. The information of conserved core genes used in the Phylogenetic trees**

| **Name** | **Accession number of proteins** |
| --- | --- |
| **Xis** | **WAB22393.1** |
| **Int** | **AAL59748.1** |
| **RumB** | **AAL59747.1** |
| **RumA** | **AAL59746.1** |
| **T4SS Relaxase MOBH** | **AAL59675.1** |
| **T4SS t4cp2** | **AAL59680.1** |
| **T4SS MPF protein *traL*** | **AAL59674.1** |
| **T4SS MPF protein *traE*** | **AAL59679.1** |
| **T4SS MPF protein *traB*** | **AAL59682.1** |
| **T4SS MPF protein *traV*** | **AAL59672.1** |
| **T4SS MPF protein *traA*** | **AAL59683.1** |
| **T4SS coupling protein *virB4*** | **AAL59681.1** |
| **T4SS MPF protein *traW*** | **AAL59671.1** |
| **T4SS MPF protein *traU*** | **AAL59673.1** |
| **T4SS MPF protein *traN*** | **AAL59755.1** |
| **Bet** | **AAL59710.1** |
| **Eox** | **AAL59709.1** |
| **T4SS MPF protein *traF*** | **AAL59678.1** |
| **T4SS MPF protein *traH*** | **AAL59676.1** |
| **T4SS MPF protein *traG*** | **AAL59677.1** |
| **Eex** | **AAL59699.1** |
| **SetC** | **AAL59693.1** |
| **SetD** | **AAL67891.1** |
| **CroS** | [**AAL59694.1**](https://www.ncbi.nlm.nih.gov/protein/21885288) |
| **SetR** | [**AAL59692.1**](https://www.ncbi.nlm.nih.gov/protein/21885286) |

**Figure S1. Linear alignment of ICE*Sal*NJT6 with other homologous sequences available in the NCBI database. The red and light yellow arrows represent the drug resistance genes and insertion sequences, respectively.**


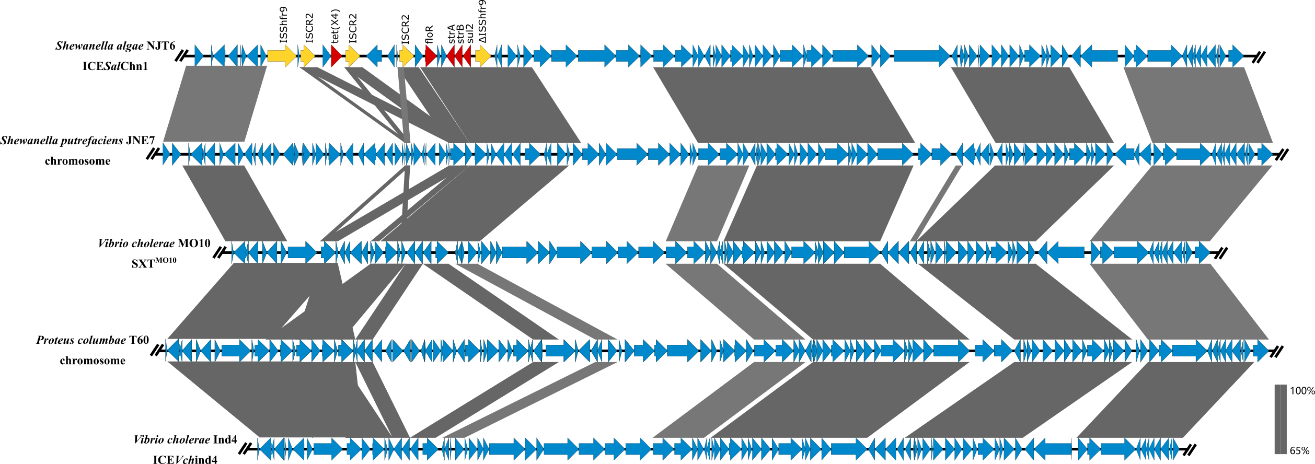


**Figure S2. Linear alignment of ICE*Sch*NTT9 with other homologous sequences available in the NCBI database.**


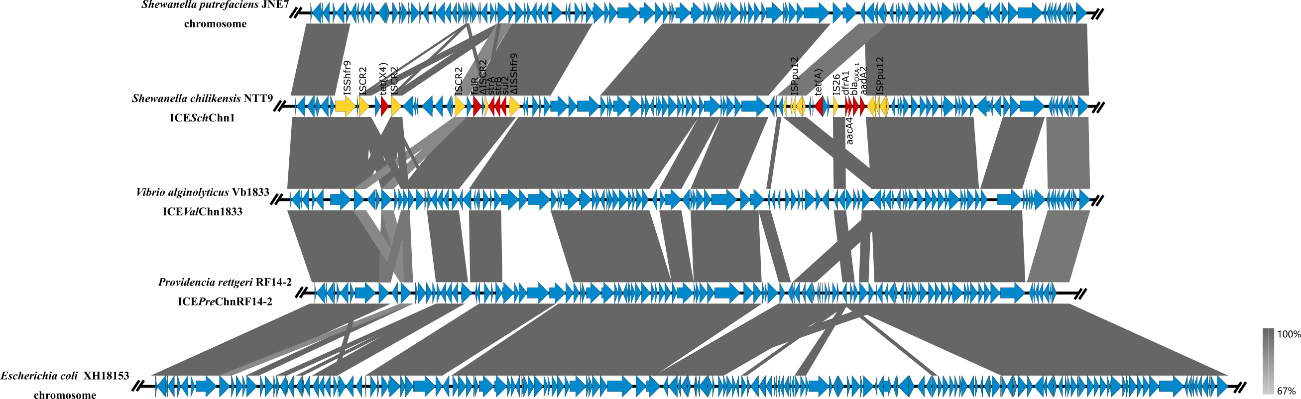


**Figure S3. Linear alignment of ICE*Sch*ST10 with other homologous sequences available in the NCBI database.**


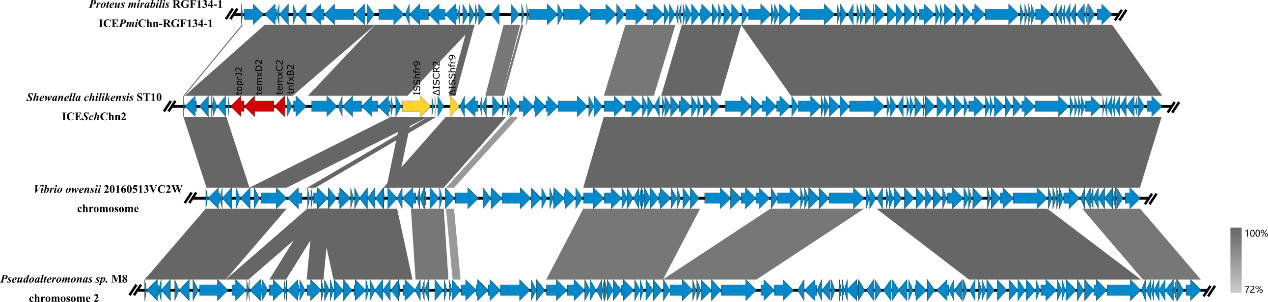


**Figure S4. Linear alignment of the VRⅢ of ICE*Sal*NJT6 and ICE*Sch*NTT9 with homologous sequences available in the NCBI database.**


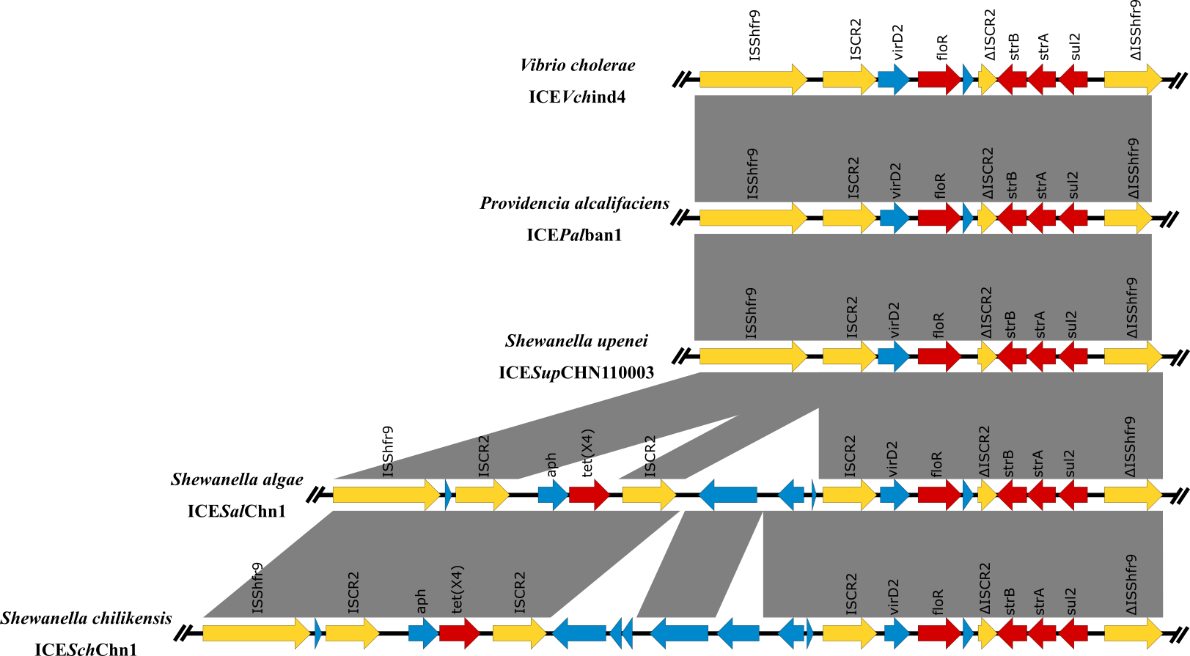


**Figure S5. Linear alignment of the SGI1-family elements in *S. chilikensis* strain ST10 with homologous sequence available in the NCBI database. The *mnmE* and *mnmG* genes are highlighted in purple.**


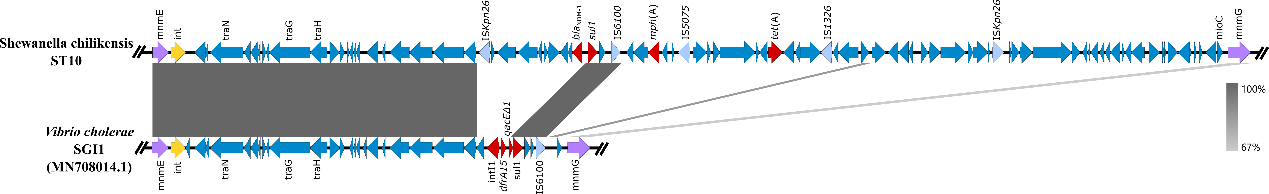


**Figure S6. Pairwise competition assay of the isolated ICEs.**


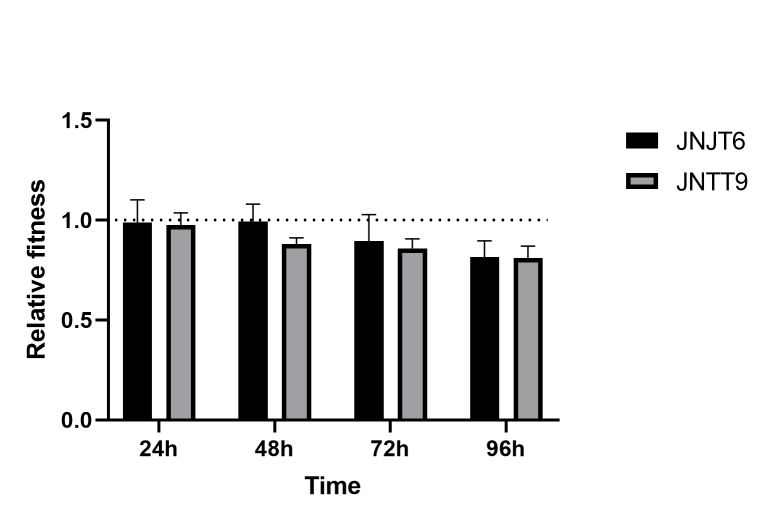


**Figure S7. Gel electrophoresis image of PCR amplicons corresponding to the circular intermediate and *prfC* after excision of ICE*Sch*ST10. P1P2 represents *prfC* gene after excision of ICE*Sch*ST10, and P3P4 represents the circular intermediate of ICE*Sch*ST10.**

**
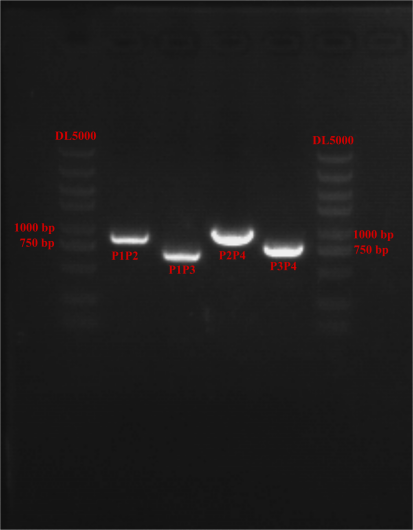
**
